# A triple-drug treatment regimen to accelerate elimination of lymphatic filariasis: From conception to delivery

**DOI:** 10.1093/inthealth/ihaa046

**Published:** 2020-12-22

**Authors:** Gary J Weil, Julie A Jacobson, Jonathan D King

**Affiliations:** Department of Medicine, Washington University School of Medicine, St. Louis, Missouri, USA; Bridges to Development, Seattle, Washington, USA; Department of Control of Neglected Tropical Diseases, World Health Organization, Geneva, Switzerland

**Keywords:** elimination, ivermectin, lymphatic filariasis, mass drug administration, therapy

## Abstract

The Global Programme to Eliminate Lymphatic Filariasis (LF) is using mass drug administration (MDA) of antifilarial medications to treat filarial infections, prevent disease and interrupt transmission. Almost 500 million people receive these medications each year. Clinical trials have recently shown that a single dose of a triple-drug combination comprised of ivermectin, diethylcarbamazine and albendazole (IDA) is dramatically superior to widely used two-drug combinations for clearing larval filarial parasites from the blood of infected persons. A large multicenter community study showed that IDA was well-tolerated when it was provided as MDA. IDA was rapidly advanced from clinical trial to policy and implementation; it has the potential to accelerate LF elimination in many endemic countries.

## Introduction

The Global Programme to Eliminate Lymphatic Filariasis (GPELF), launched by the World Health Organization (WHO) in 2000, aims to eliminate lymphatic filariasis (LF) as a public health problem.^1^ The Programme has two pillars, namely mass drug administration (MDA) with antifilarial drugs (to cure infections and reduce transmission of new infections) and protocols for morbidity management and disability prevention (MMDP) to help persons with clinically overt LF (lymphedema, elephantiasis and hydrocele). Other papers in this collection describe GPELF and the double-drug regimens (ivermectin plus albendazole and diethylcarbamazine (DEC) plus albendazole) that have been used for MDA since its inception. While those treatments have strong antifilarial activity, neither results in complete cures (death of all adult worms) or achieves sustained, complete clearance of microfilaremia (Mf) in a majority of infected persons after a single dose. Long-lasting Mf clearance is desirable, because blood Mf are required for transmission of new filarial infections by mosquitoes. This paper will review clinical development of the triple-drug regimen IDA that is comprised of ivermectin plus diethylcarbamazine and albendazole; the review process for policy change; and IDA’s rapid uptake by LF elimination programs.

## A triple-drug MDA regimen for LF elimination

The DOLF Project is a large consortium for research on selected neglected tropical diseases that was funded by the Bill & Melinda Gates Foundation in 2010 (www.dolf.wustl.edu). One of the major objectives of DOLF was to conduct clinical trials and community treatment studies to optimize use of approved drugs to try to improve the impact of MDA to accelerate elimination programs for LF and onchocerciasis. DOLF plans research projects with input from key stakeholders. It also provides technical support for field studies that are conducted by colleagues and staff from health ministries and/or academic institutions in disease-endemic countries.

While the idea of triple-drug therapy for LF was not novel, DOLF conducted the first clinical trials that compared the tolerability and efficacy of IDA (a single oral dose of ivermectin 200 μg/kg plus 6 mg/kg of DEC and 400 mg of albendazole) to two-drug MDA regimens that are recommended by WHO, namely DEC plus albendazole (DA) and ivermectin plus albendazole (IA).

Preliminary pharmacokinetic studies showed that there were no clinically significant interactions between the three drugs.^2^ Clinical trials in heavily infected individuals showed that IDA was more effective than DA for achieving sustained clearance of *Wuchereria bancrofti* Mf in Papua New Guinea (PNG); 96% of IDA recipients were Mf negative three years after a single dose.^2,3^ Follow-up studies showed that most persons in this trial remained amicrofilaremic when they were retested almost five years after treatment with a single dose of IDA.^4^ Similar results (albeit with shorter follow-up) were obtained in a clinical trial of IDA that was performed in persons with *Brugia timori* infections in eastern Indonesia; 96% of persons treated with IDA had complete Mf clearance by membrane filtration one year after IDA treatment (T. Supali, personal communication). A clinical trial in Côte d'Ivoire showed that IDA was much more effective for clearing *W. bancrofti* Mf and more effective for killing adult filarial worms than IA, but the Mf clearance effect was not as long-lasting as that seen in the studies in PNG or Indonesia.^5^ Seventy-one percent of IDA recipients were Mf negative by membrane filtration at 12 months compared to 26% after ivermectin plus albendazole. However, a single treatment with IDA was equivalent to two annual treatments with IA in that setting. Additional work will be needed to determine whether reappearance of Mf in trial participants in Côte d'Ivoire was due to reinfections after IDA treatment or to a regional difference in parasite susceptibility to IDA treatment.

## Steps that led to policy change

Transmission modeling studies suggested that MDA with IDA (with moderate to high compliance) should reduce the number of rounds of MDA required to eliminate LF (defined in the study as achieving a true Mf prevalence of <1%) compared to the number of rounds that would be required to reach the elimination threshold with the two-drug MDA regimens.^6^ External stakeholders advised the DOLF team that additional evidence would be required before IDA could be recommended for widespread use in LF elimination programs. Tolerability is a top priority for MDA programs, and the small clinical trials (conducted in heavily infected subjects) had shown that mild to moderate adverse events were sometimes more common after IDA treatment than after DA or IA. Clearly, additional evidence was needed to assess the tolerability of IDA in community settings. WHO advised assessing the frequency of adverse events in communities receiving IDA MDA through a strategy of cohort event monitoring.^7^ Therefore, DOLF researchers conducted large-scale community studies that compared the prevalence and severity of AEs following MDA with either IDA or DA.^8^ The studies were performed in carefully selected sites in five countries with different epidemiological characteristics. Three studies were performed in *W. bancrofti-*endemic areas (in India, Fiji and Haiti) that had persistent LF despite five or more prior rounds of MDA with DA. Two studies were performed in treatment-naïve areas in PNG and Indonesia that were endemic for *W. bancrofti* and *B. timori*, respectively. Results from more than 26 000 participants showed that overall AE rates, types and severity were the same after IDA and DA.^8^ AE rates were higher after IDA than after DA in persons with microfilaremia, but severe or serious AEs were no more common after IDA than after DA. These results indicated that IDA was as well-tolerated as DA for use in MDA programs. The large dataset from this study (which included age, height and weight) was used to develop a practical height-based dosing algorithm to decrease the frequency of persons receiving less than the recommended dose of DEC that occurs with age-based dosing.^9^

A more effective treatment regimen for MDA will not improve outcomes unless it is acceptable to target populations. Therefore, acceptability studies were performed in each of the five tolerability study sites several months after MDA. These studies utilized a common protocol that included a survey questionnaire, focus group sessions and key informant interviews.^10^ Results of these studies showed that IDA was as acceptable as DA even though the IDA regimen contains more tablets. The study identified factors that are likely to affect the acceptability of MDA with any regimen. These included a well-planned and executed social mobilization program and factors that increased trust in the program (e.g. clearly identifiable drug distributors who were well-informed, and a transparent plan for managing AEs that sometimes arise following treatment). Lessons from these acceptability studies and lessons learned from the first countries that have employed IDA are being used to inform large-scale introduction of IDA in a number of countries.

## Policy changes at WHO and at MSD

One unique aspect of the transition of IDA research findings to policy was the close coordination and communication between stakeholders with WHO. The WHO guideline development process was followed and timed appropriately to reduce lag times between discovery, evidence review and policy development.^11^ WHO obtained approval to initiate development of a new guideline on MDA in late 2016 based on exciting (but unpublished at that time) 24-month follow-up data from the IDA PK study and 12-month data from a larger randomized control trial in PNG. After establishing the scope of the new guideline, WHO commissioned systematic reviews to address the specific PICO (patient population, interventions, comparisons and outcomes) questions to guide formulation of recommendations. All data from relevant studies (published and unpublished from DOLF's registered clinical trials) were shared by researchers and included in the systematic review and meta-analysis. The Guideline Development Group reviewed the evidence (efficacy, tolerability and acceptability data) and used GRADE procedures (Grading of Recommendations Assessment, Development and Evaluation) to formulate recommendations regarding alternative drug regimens for MDA.^12^ The group also considered factors such as resources, health equity and feasibility; this process led to a conditional recommendation for the use of IDA in certain settings. Full details of the process and recommendations on other alternative MDA strategies were published in the new guideline on November 17, 2017.^13^ Briefly, the policy change endorsed use of IDA for LF elimination programs in countries without onchocerciasis that had either not yet started MDA or that were not on track to reach elimination targets following several rounds of MDA with DA. Shortly following the release of the guideline, MSD (trade name of Merck & Co. Inc., Kenilworth, NJ, USA) announced a substantial increase in their ivermectin donation program of up to 100 million treatments per year for five years through their Mectizan Donation Programme to cover projected needs for IDA in countries without co-endemic onchocerciasis.^14^ This remarkable outcome was possible because MSD had been informed and engaged early in the process. Indeed, all study data and modeling results were shared with MSD to inform their internal decision-making process.

## Uptake of the new policy by national LF-elimination programs

Even prior to the release of the new guideline, WHO Regional Offices received requests from endemic countries for a recommendation on IDA and also requests for WHO to recommend alternative MDA strategies that could accelerate progress toward LF elimination.^15^ Immediately following the release of the guideline, WHO organized regional or country-level consultations to review progress, identify where IDA was warranted and plan according to the decisions by national programs to adopt IDA. These meetings also provided opportunities for renewed engagement among stakeholders in support of the national programs’ plans. The first program implementation of IDA occurred in the Pacific region when Samoa and American Samoa implemented IDA MDA in 2018. The same year, Kenya, Fiji, India and PNG planned and implemented IDA in pilot districts.^16^ In 2019, Guyana, Malaysia, Sao Tome and Principe, Timor Leste and Tuvalu adopted IDA nationally by providing IDA in all implementation units (IU) that required MDA. Egypt was the first country to use IDA in a community setting to address focal persistence of LF that was detected by post-elimination surveillance. As of May 2020, IDA MDA has already been provided to more than 13 million people in targeted implementation units in 11 countries to remove the threat of LF transmission and prevent new infections (WHO unpublished data).

## Research questions and future directions

IDA worked better than expected in the clinical trials, and that was a wonderful surprise. The success of IDA and the roll-outs have raised additional questions for scientists and for LF elimination programs:

How does IDA work? We do not know why IDA is more effective than IA or DA for clearing Mf. Ultrasound results from clinical trials suggest that IDA is more effective than IA for killing adult worms,^5^ but antigen test results suggest that IDA is no more effective than DA for killing adult worms. Therefore, it is likely that IDA kills some adult worms and sterilizes others. Additional work is needed to determine whether this is due to additive effects or to synergy between IDA components. Since anti-*Wolbachia* treatments are more effective for sterilizing than killing adult filarial worms,^17^ it is possible that IDA's sterilizing effect on adult worms is secondary to an indirect effect of the treatment on *Wolbachia* endosymbionts.

Are there regional differences in IDA efficacy? While IDA has worked well in many areas, researchers have noted reduced efficacy of IDA for achieving sustained clearance of Mf in some areas. More work is needed to determine whether this is due to reinfection or to differences in drug levels or in parasite susceptibility to the treatment.

Implications of IDA for MDA stopping decisions. Clinical trials and community MDA studies have shown that IDA is much more effective for clearing Mf than for clearing CFA. Current WHO protocols for transmission assessment surveys (TAS) and pre-TAS rely heavily on CFA testing that is performed after at least five rounds of MDA with two-drug regimens.^18^ Experts are currently reevaluating these protocols, because CFA prevalences in children or adults are unlikely to decrease sufficiently to meet current targets after two rounds of IDA. Since Mf are required for LF transmission, programs should consider going back to the future in areas where IDA is used, by basing stopping decisions on a modified version of Mf monitoring. A two-step process could start with CFA screening of adults to reduce the number of persons who require Mf testing.

Can IDA be safely deployed in African countries with loiasis or onchocerciasis? WHO does not recommend use of IDA in these countries, because DEC can cause serious adverse events in persons with heavy infections with *Loa loa* or *Onchocerca volvulus*. The DOLF project is currently conducting a clinical trial to determine whether IDA can be safely used in persons with onchocerciasis after skin and intraocular Mf counts have been reduced by pretreatment with ivermectin alone.

Will IDA work for other filarial infections (especially onchocer-ciasis)? This is an interesting and important question that is also being addressed in the clinical trial mentioned above. *O. volvulus* worms are more difficult to kill than *W. bancrofti*. However, a well-tolerated treatment that permanently sterilizes adult *O. volvulus* would be a game changer for the global programme to eliminate onchocerciasis.

Off-target impacts of IDA. Ivermectin had not been widely used in Asia prior to IDA, because MSD's donation had historically focused on Africa. Research is needed to document the health impacts of IDA on ectoparasitic infections, STH and strongyloidiasis, and to assess whether these benefits or marketing of IDA as a new and improved MDA regimen can improve compliance for LF elimination programs.

Ivermectin supply. The use of IDA in all areas where warranted according to current WHO guidelines (especially in Asia) would require ivermectin beyond the committed donation. In such a scenario, additional sources of quality-assured ivermectin will need to be identified.

## Lessons from the IDA case study for accelerated uptake of research advances by global health programs

Medical research too often focuses on discovery as an end in itself. The IDA story illustrates what can happen when partners coordinate their efforts with a focus on the global public health benefit. Close collaboration and partnership compressed the time required to move from research advance to policy change and implementation. A process that normally may have taken more than 10 years was completed in approximately three years. We believe that early communication, coordination between key stakeholders and a focus on steps required for policy change were the special sauce that led to this success. This collaboration started early in the DOLF project with close communication between the researchers and the funding agency. The researchers contacted the funding agency to report the dramatic results from the pilot IDA study in PNG. They convened their project's technical advisory group (TAG) to share early results from a larger study in PNG, and the TAG endorsed their plan to conduct an additional IDA clinical trial for LF in Africa to attempt to confirm the exciting results that were coming out of PNG. The funding agency's flexible, adaptive grant management policy provided resources needed to generate the additional data. The Côte d'Ivoire study design was modified so that six-month efficacy data from that country could be considered together with longer-term efficacy data that was anticipated soon from PNG.

Proactive planning identified key stakeholders that would need to be involved for discussions if the new clinical studies confirmed early results from the first IDA clinical trials. A broad stakeholder meeting was convened as soon as the clinical data were available. That meeting led to a research plan designed to provide evidence needed for policy change. A large, multicenter tolerability study with a strong acceptability assessment component was one of the most important action items, and the funding agency readily agreed to support this critical study. These steps were coupled with a communication plan that included disease-endemic countries, donor organizations, pharmaceutical partners and regulatory agencies to facilitate decision-making and subsequent adoption of new policy recommendations. This early communication resulted in prompt consideration of the new WHO guideline by endemic countries and by donors who were willing to support introduction of IDA, an expanded ivermectin donation program and new research projects to monitor IDA impact and acceptability.

## Conclusions

Clinical trials demonstrated that IDA is well-tolerated and more effective for clearing microfilaremia than prior treatments for LF. The fact that all components of IDA were previously approved drugs facilitated the rapid development and acceptance of the combination treatment. IDA has the potential to accelerate elimination of LF in countries that are not coendemic for onchocerciasis or loiasis. The IDA case study contains important lessons for those who are keen to accelerate the transition of research advances into new policies that can be implemented to benefit patients and populations.
